# Evidence map for randomized controlled trials of treatment of acupuncture and moxibustion for dry eye disease

**DOI:** 10.3389/fmed.2026.1829981

**Published:** 2026-04-21

**Authors:** Qidi Liu, Xiaobing Yang, Yanyan Hong, Tingting Wang, Sixuan Han

**Affiliations:** 1 School of Nursing, Nanjing University of Chinese Medicine, Nanjing, China; 2 Department of Nursing, Nanjing Hospital of Chinese Medicine, Affiliated to Nanjing University of Chinese Medicine, Nanjing, China

**Keywords:** acupuncture, dry eye disease, evidence map, moxibustion, randomized controlled trial

## Abstract

**Objective:**

This study systematically integrated the clinical evidence on acupuncture and moxibustion treatments for dry eye disease through evidence mapping, with the aim of providing more intuitive and reliable evidence-based support for the clinical management of dry eye disease with acupuncture and moxibustion.

**Methods:**

Randomized controlled trials (RCTs) on acupuncture and moxibustion for dry eye disease published from database inception to December 1, 2025 were retrieved from China National Knowledge Infrastructure (CNKI), VIP Database, WanFang Data, China Biology Medicine disc (CBM), PubMed, Web of Science, Embase, and the Cochrane Library. The methodological quality of the included RCTs was evaluated using the Risk of Bias 2.0 (RoB 2.0) tool. An evidence map was used to present the basic characteristics, interventions, outcome indicators, and quality assessment results of the included studies.

**Results:**

A total of 319 studies were included. Six traditional Chinese medicine (TCM) syndrome types were involved, among which liver-kidney yin deficiency syndrome was the most common, accounting for 54.55%. Regarding interventions, 17 types of monotherapy and 31 types of combination therapy were identified. Outcome indicators were classified into 13 categories, with the top three most frequently used being ocular surface health assessment indicators, TCM symptom scores, and clinical efficacy. Overall, the methodological quality of the included literature was generally low.

**Conclusion:**

Acupuncture and moxibustion show certain advantages in the treatment of dry eye disease; however, the methodological design of existing RCTs still requires improvement. The application of TCM-related outcome indicators in dry eye disease assessment, as well as the standardized use of TCM syndrome differentiation, also need to be strengthened. Future studies should strictly adhere to randomization requirements and appropriately handle data to provide higher-level evidence supporting acupuncture and moxibustion for dry eye disease.

## 1 Introduction

Dry eye disease is the most common chronic ocular surface disorder encountered in clinical practice, characterized by symptoms such as dryness, foreign body sensation, photophobia, blurred vision, and visual fatigue (1). Its major risk factors include female sex, advanced age, Asian ethnicity, excessive use of electronic devices, and worsening environmental pollution (2, 3). Epidemiological investigations have shown that the global prevalence of dry eye disease ranges from 5 to 50%, while in China it ranges from 20 to30% (4, 5), making it one of the most prevalent ocular surface diseases in clinical settings. Dry eye disease may also lead to anxiety, depression, and other psychological problems, thereby seriously impairing patients’ quality of life and physical and mental health, while imposing a substantial socioeconomic burden (6).

At present, artificial tears are the first-line therapy for dry eye disease. By supplementing exogenous tears to lubricate the ocular surface, they can relieve dry eye symptoms in the short term; however, they lack stable and sustained efficacy (7). In addition, most artificial tear preparations contain preservatives, which may cause tear film instability, corneal damage, and other adverse effects (8). As a common non-pharmacological therapy in traditional Chinese medicine, acupuncture and moxibustion can significantly improve indicators such as tear film break-up time and Schirmer test results, thereby providing new perspectives and therapeutic approaches for the treatment of dry eye disease (9, 10). Randomized controlled trials (RCTs) represent the highest level of evidence among primary studies and constitute the most critical basis for the development of clinical practice guidelines. However, a systematic synthesis of the evidence in this field remains insufficient.

Evidence mapping was first proposed in the field of evidence-based medicine in 2003. Through the systematic analysis of research findings in a specific field, it visually presents the distribution characteristics of available evidence and thereby reveals directions for future research development (11–13). By integrating RCTs, this study aims to provide a more scientific reference framework for future clinical research on acupuncture and moxibustion interventions for dry eye disease.

## 2 Methods

### 2.1 Literature search

RCTs on acupuncture and moxibustion for dry eye disease were searched in China National Knowledge Infrastructure (CNKI), VIP Database, WanFang Data, China Biology Medicine (CBM), PubMed, Web of Science, Embase, and the Cochrane Library from database inception to December 1, 2025. The search terms included “Acupuncture,” “Acupuncture therapy,” “Acupuncture Points,” “Acupuncture, Ear,” “Moxibustion” and “Dry Eye Disease” (Supplementary File 1).

### 2.2 Inclusion and exclusion criteria

Inclusion criteria:

1. Study type: RCTs on acupuncture and moxibustion for dry eye disease.
2. Participants: patients clinically diagnosed with dry eye disease.
3. Interventions: the experimental group received acupuncture and/or moxibustion as the main intervention, either alone or in combination with other traditional Chinese medicine or conventional Western medicine treatments; the control group received conventional Western medicine treatment, placebo, or no treatment.

Exclusion criteria:

1. Duplicate publications, in which case only the study with the most complete information was retained.
2. Studies with ineligible designs, including animal experiments, reviews, meta-analyses, systematic reviews, case reports, conference papers, and data mining studies.
3. Studies for which the full text was unavailable.
4. Studies published in languages other than Chinese or English.

### 2.3 Study selection and data extraction

Two researchers (Qidi Liu and Xiaobing Yang) independently performed the literature screening. Duplicate records were first removed using NoteExpress software, after which titles and abstracts were screened for preliminary eligibility. Full texts of potentially eligible studies were then reviewed for final inclusion. Any disagreements were resolved through discussion with a third researcher (Sixuan Han). The extracted data included the study title, publication year, authors, sample size, intervention measures, and outcome indicators.

### 2.4 Methodological quality assessment

The methodological quality of the included RCTs was assessed using the Risk of Bias 2.0 (RoB 2.0) tool recommended by the Cochrane Handbook. Five domains were evaluated: bias arising from the randomization process, bias due to deviations from intended interventions, bias due to missing outcome data, bias in the measurement of outcomes, and bias in the selection of the reported result. An overall risk of bias judgment was then made based on the assessments across all domains, with each item rated as “Low risk,” “Some concerns,” or “High risk.” The assessment was conducted independently by two researchers (Qidi Liu and Xiaobing Yang) and cross-checked for consistency. Any disagreements were resolved through discussion with a third researcher (Tingting Wang).

### 2.5 Data analysis

As this study was designed as an evidence map, no meta-analysis was performed. The primary aim was to systematically summarize and visually present the distribution characteristics of the existing clinical evidence on acupuncture and moxibustion for dry eye disease. Microsoft Excel 2022 was used to present sample size, treatment duration, traditional Chinese medicine syndrome types, interventions, and outcome indicators in tabular form. However, after data synthesis and analysis, substantial heterogeneity was observed across these aspects, which limited the feasibility of conducting a meaningful quantitative synthesis. Therefore, descriptive analysis and evidence mapping were used to summarize the included studies. GraphPad Prism 10.1.2 was used to generate the publication bias plot and bubble charts.

## 3 Results

### 3.1 Study selection process and results

**Figure 1:**
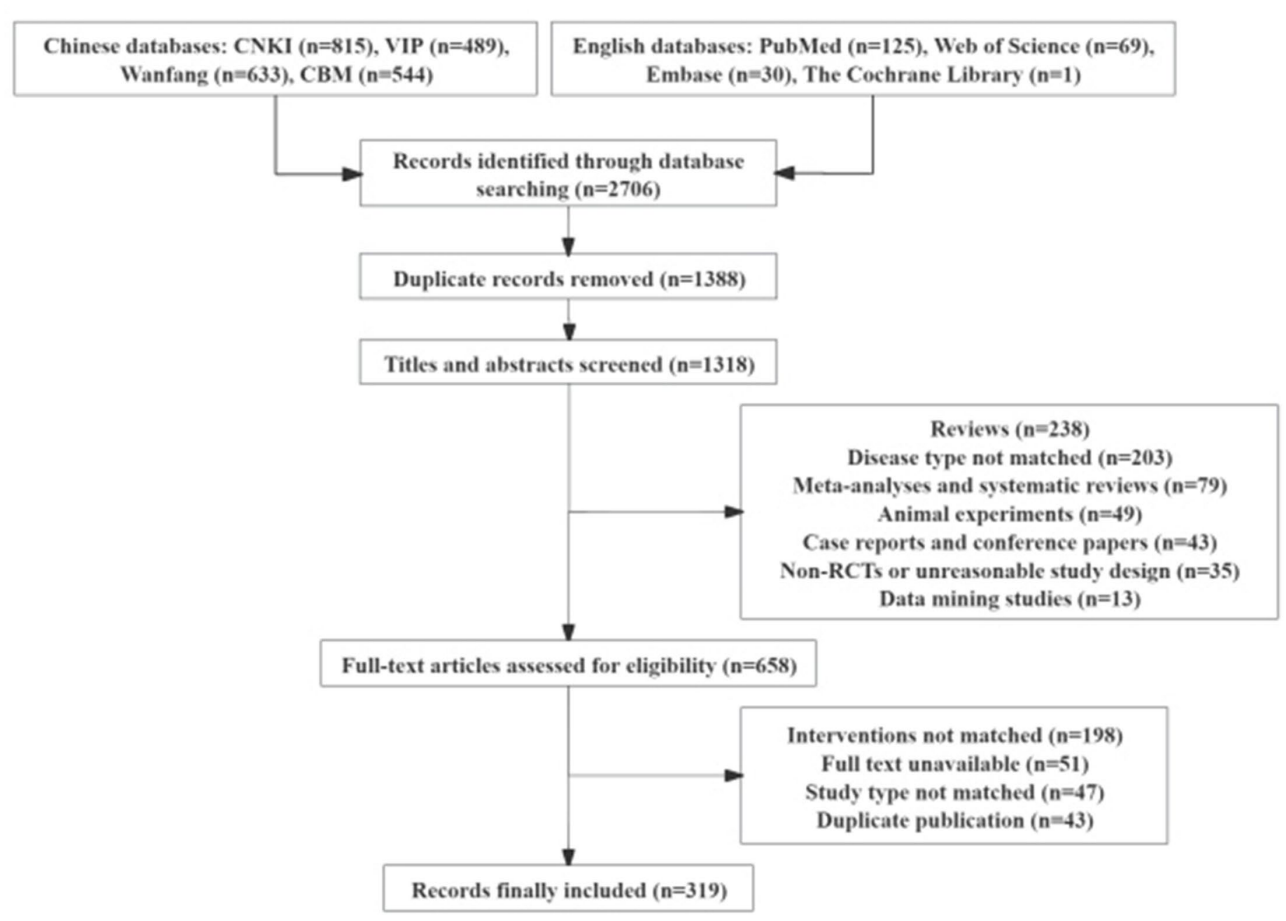
Flowchart of the literature selection process for studies on acupuncture and moxibustion for dry eye disease.

A total of 2,706 records were identified through the search strategy, including 2,481 Chinese-language articles and 225 English-language articles. After removing duplicates, 1,318 records remained. Following title and abstract screening, 653 records were excluded. After full-text assessment, 339 studies were further excluded, resulting in the inclusion of 319 RCTs. The study selection process is presented in Figure 1.

### 3.2 Publication types and trends

**Figure 2:**
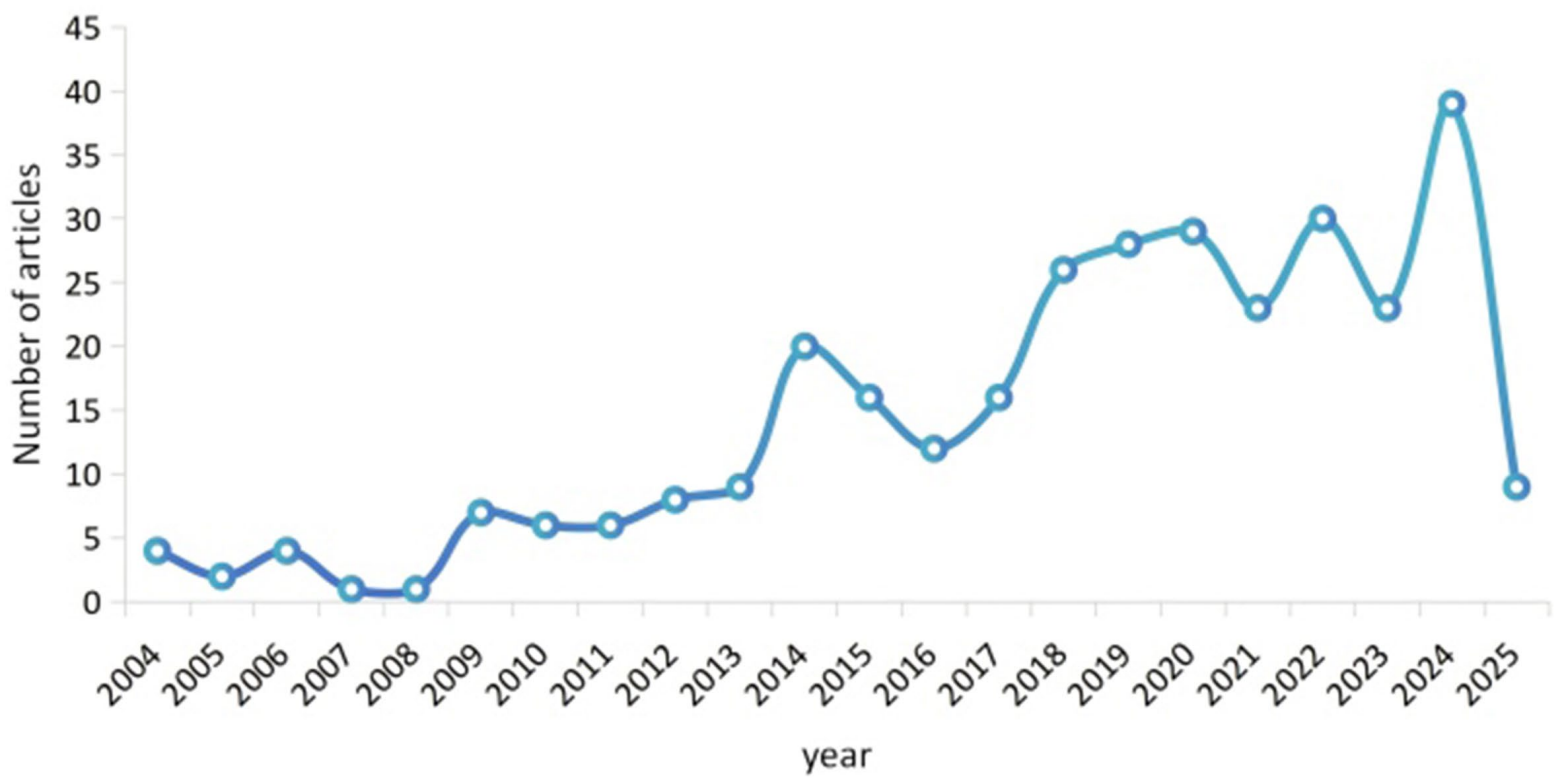
Publication trends of studies on acupuncture and moxibustion for dry eye disease.

Of the 319 included RCTs, 303 were published in Chinese and 16 in English. In terms of publication type, 260 were journal articles and 59 were dissertations or theses. Regarding publication trends, the annual number of publications increased gradually before 2014, with fewer than 21 studies published each year. From 2016 to 2020, the number of publications in this field showed a continuous upward trend, suggesting that research on dry eye disease was in a period of growing interest. Over the past 5 years, the number of publications fluctuated and reached a peak in 2024. The publication trend is shown in Figure 2.

### 3.3 Sample size

Among the included RCTs, the sample size ranged from 18 to 640 participants. Studies with sample sizes of 60 to 100 participants were the most common (194 studies, 59.51%), while only 4 studies included 300 or more participants. Details are presented in Table 1.

**Table 1:** Sample sizes of studies on acupuncture and moxibustion interventions for dry eye disease.

Sample sizes	Number of articles	Percentage (%)
<60	53	16.26
60 ~ 100	194	59.82
100 ~ 200	72	22.09
200 ~ 300	3	0.92
≥300	4	1.23

### 3.4 Intervention duration

Among the 319 included RCTs, 253 reported the intervention duration, whereas 66 did not provide a description of the treatment duration. The shortest intervention duration was 1 week and the longest was 12 weeks. Studies with an intervention duration of 4 weeks were the most common (144 studies, 45.14%). Details are shown in Table 2.

**Table 2:** Duration of acupuncture and moxibustion interventions for dry eye disease.

Intervention duration	Number of articles	Percentage (%)
4 weeks	144	45.14
3 weeks	38	11.91
2 weeks	23	7.21
8 weeks	19	5.96
20 days	8	2.51
12 weeks	6	1.88
10 days	5	1.57
5 weeks	4	1.25
1 week	2	0.63
12 days	2	0.63
9 weeks	1	0.31
6 weeks	1	0.31

### 3.5 Intervention measures

Among the RCTs on acupuncture and moxibustion for dry eye disease, 17 types of monotherapy were identified, including 11 acupuncture-based therapies and 6 moxibustion-based therapies; 31 types of combination therapy were also identified, yielding a total of 48 intervention types. Among the monotherapies, manual acupuncture was the most frequently reported intervention (140 studies, 44.89%). Among the combination therapies, manual acupuncture combined with oral Chinese herbal medicine was the most common approach (56 studies, 17.55%). Details are presented in Table 3.

**Table 3:** Intervention measures of acupuncture and moxibustion for dry eye disease.

Number	Intervention measures	Number of articles	Percentage (%)
1	Manual acupuncture	140	43.89
2	Electroacupuncture	9	2.82
3	Eye acupuncture	3	0.94
4	Press needle	2	0.63
5	Fire needle	2	0.63
6	Scalp acupuncture	2	0.63
7	Floating acupuncture	1	0.31
8	Plum-blossom needle	1	0.31
9	Abdominal acupuncture	1	0.31
10	Body acupuncture	1	0.31
11	Acupoint injection	1	0.31
12	Thunder-fire moxibustion	8	2.51
13	Walnut-shell moxibustion	3	0.94
14	Warm needle moxibustion	1	0.31
15	Moxibustion	1	0.31
16	Heat-sensitive moxibustion	1	0.31
17	Moxibustion during motion	1	0.31
18	Manual Acupuncture + Oral herbal medicine	56	17.55
19	Manual Acupuncture + External herbal therapy	21	6.58
20	Manual Acupuncture + Thunder-fire moxibustion	10	3.13
21	Manual Acupuncture + Moxibustion	3	0.94
22	Manual Acupuncture + Meibomian gland massage	3	0.94
23	Manual Acupuncture + External herbs + Oral herbs	3	0.94
24	Manual Acupuncture + Warm needle moxibustion	2	0.63
25	Manual Acupuncture + Acupoint massage	2	0.63
26	Manual Acupuncture + Auricular taping	1	0.31
27	Manual Acupuncture + Bloodletting	1	0.31
28	Manual Acupuncture + Walnut-shell moxibustion	1	0.31
29	Manual Acupuncture + Thunder-fire moxibustion + Auricular taping	1	0.31
30	Manual Acupuncture + Heat-sensitive moxibustion	1	0.31
31	Manual Acupuncture + Acupoint application	1	0.31
32	Manual Acupuncture + Acupoint injection	1	0.31
33	Manual Acupuncture + External herbs + Meibomian massage	1	0.31
34	Electroacupuncture + Thunder-fire moxibustion + External herbs	1	0.31
35	Electroacupuncture + External herbal therapy	1	0.31
36	Electroacupuncture + Oral herbs + External herbs	1	0.31
37	Electroacupuncture + External herbal therapy	1	0.31
38	Press needle + Auricular seed taping	1	0.31
39	Press needle + Oral herbal medicine	1	0.31
40	Press needle + External herbal therapy	1	0.31
41	Eye acupuncture + Oral herbal medicine	1	0.31
42	Thunder-fire moxibustion + External herbal therapy	7	2.19
43	Thunder-fire moxibustion + Oral herbal medicine	6	1.88
44	Thunder-fire moxibustion + Meibomian gland massage	5	1.57
45	Thunder-fire moxibustion + Acupoint massage	4	1.25
46	Moxibustion + Bloodletting	1	0.31
47	Moxibustion + External herbal therapy	1	0.31
48	Thunder-fire moxibustion + Auricular therapy	1	0.31

### 3.6 Analysis of traditional Chinese medicine syndrome types

**Figure 3:**
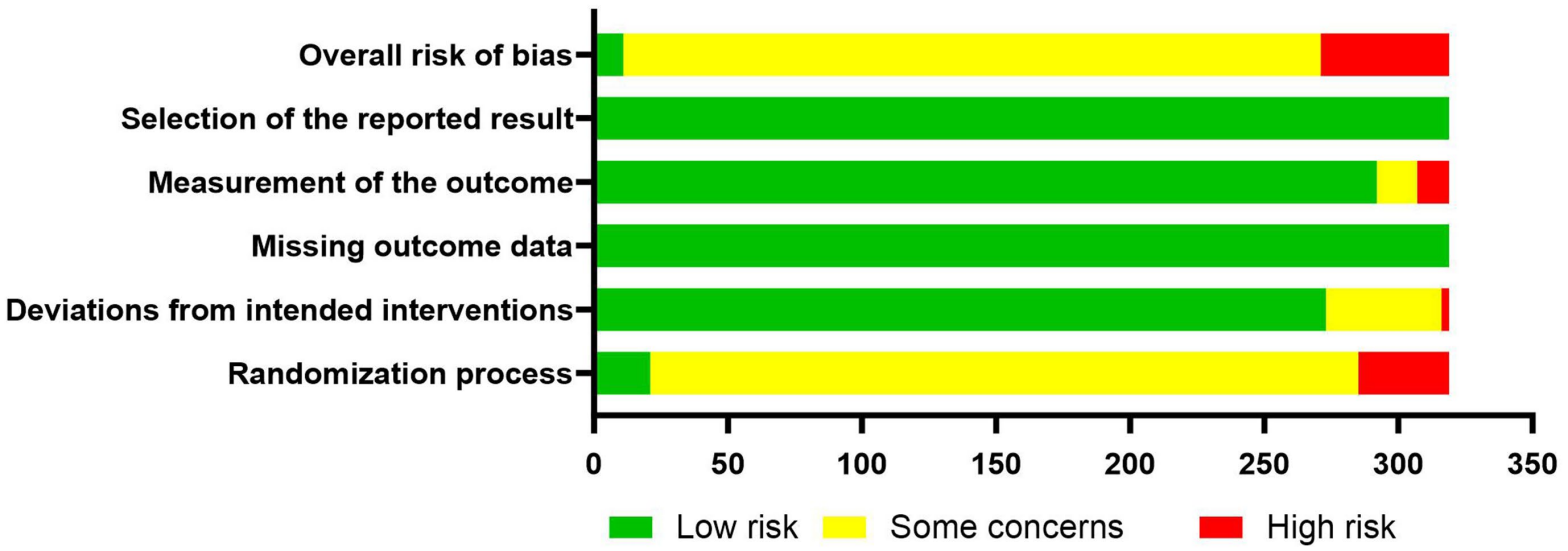
Risk-of-bias assessment of randomized controlled trials on acupuncture and moxibustion for dry eye disease.

Among the 319 included RCTs, 77 studies reported traditional Chinese medicine TCM syndrome differentiation, whereas 242 did not mention TCM syndrome classification. Of the 77 studies, 68 involved a single syndrome type and 9 involved multiple syndrome types. In the present study, only studies with a single syndrome type were included in the statistical analysis. Liver-kidney yin deficiency syndrome was the most frequently reported syndrome type (42 studies, 54.55%), as shown in Table 4. In the evidence map, only studies with a single syndrome type were included for analysis. In the bubble plot, the x-axis represents different TCM syndrome types of dry eye disease, the y-axis represents different intervention measures, and the bubble size reflects the number of publications, as shown in Figure 3.

**Table 4:** Analysis of TCM syndrome types in dry eye disease.

TCM syndrome	Number of articles	Percentage (%)
Liver-kidney yin deficiency	42	54.55
Lung yin deficiency	9	11.69
Stagnant heat in liver channel	9	11.69
Qi-yin deficiency	6	7.79
Qi deficiency with blood stasis	1	1.30
Yin deficiency with dampness-heat	1	1.30

### 3.7 Outcome indicators of intervention measures

**Figure 4:**
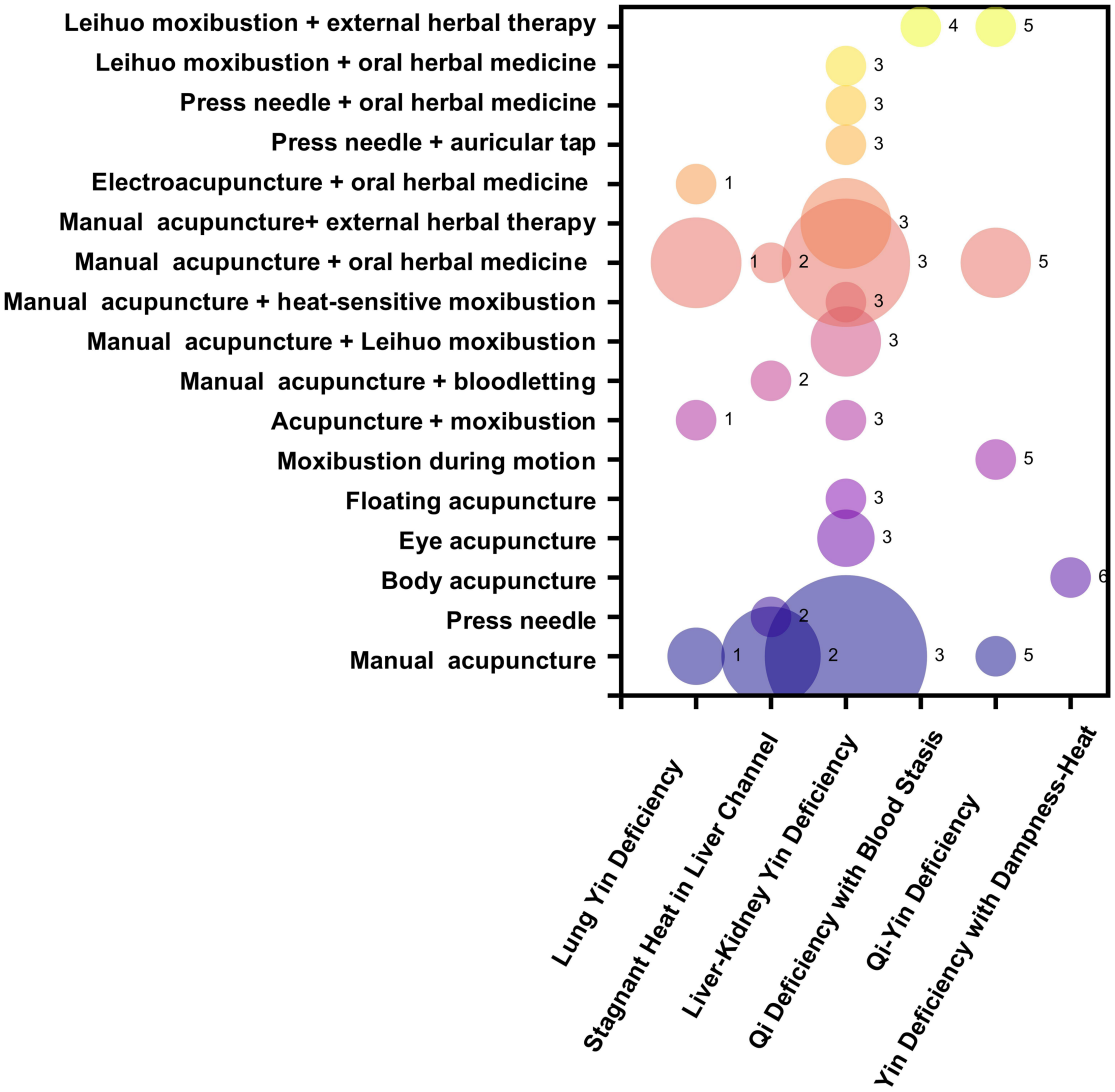
Distribution of traditional Chinese medicine syndrome types involved in acupuncture and moxibustion.

By summarizing the outcome indicators used in studies of acupuncture and moxibustion for dry eye disease, 13 categories were identified. Ranked by number of publications from highest to lowest, these categories were ocular surface health assessment indicators (295 studies), clinical efficacy (174 studies), ocular symptom and function questionnaires (113 studies), TCM symptom scores (84 studies), other indicators (75 studies), TCM syndrome scores (38 studies), inflammatory factor indicators (18 studies), psychological status (15 studies), quality of life (13 studies), immune defense biomarkers (7 studies), pain scores (5 studies), hormone indicators (4 studies), and sleep status (2 studies). The classifications and specific details are shown in Table 5. In the evidence map, the x-axis of the bubble plot represents different outcome indicators, the y-axis represents different intervention measures, and the bubble size reflects the number of publications, as shown in Figure 4.

**Table 5:** Outcome indicators of acupuncture and moxibustion interventions for dry eye disease.

Number	Types of outcome Indicators	Specific outcome indicators	Number of articles	Percentage (%)
1	Ocular surface health assessment indicators	Tear breakup time (BUT), Schirmer I test (SIT), tear meniscus height (TMH), fluorescein staining (FL), corneal fluorescein staining (CFS), surface regularity index (SRI), Intraocular Pressure, best-corrected visual acuity (BCVA), bright visual persistence (BVP), modified Meibomian Gland Score (mMGS), demodex folliculorum count	295	92.48
2	Clinical efficacy	Markedly effective rate, effective rate, ineffective rate	174	54.55
3	Ocular symptom and function questionnaires	Ocular surface disease index (OSDI), standard patient evaluation of eye dryness (SPEED), national eye institute visual function questionnaire-25 (NEI-VFQ 25), visual analog scale (VAS), ocular symptom score	113	35.42
4	TCM symptom scores	/	84	26.33
5	Other indicators	Safety assessment, occurrence of adverse events, treatment satisfaction, patient satisfaction with nursing care, acceptability assessment of acupuncture, ocular tolerability assessment, incidence of secondary bacterial infection	75	23.51
6	TCM syndrome scores	/	38	11.91
7	Inflammatory factor indicators	IL-1β, IL-4, IL-6, IL-8, IL-10, IL-13, CX3CL1, CXCL10, CXCR3, TNF-α, mmp-9	18	5.64
8	Psychological status	Hospital anxiety and depression scale (HADS), Hamilton anxiety rating scale (HAMA), Hamilton depression rating scale (HAMD), self-rating anxiety scale (SAS), self-rating depression scale (SDS), generalized anxiety disorder-7 (GAD-7), patient health questionnaire-9 (PHQ-9)	15	4.70
9	Quality of life	Short form-36 health survey (SF-36), quality of life score (QOL)	13	4.08
10	Immune defense biomarkers	Tear lactoferrin (LF)	7	2.19
11	Pain scores	Numeric rating scale (NRS)	5	1.57
12	Hormone indicators	Estradiol (E2), follicle-stimulating hormone (FSH), luteinizing hormone (LH)	4	1.25
13	Sleep status	Pittsburgh sleep quality index (PSQI)	2	0.63

### 3.8 Quality assessment of the included RCTs

**Figure 5:**
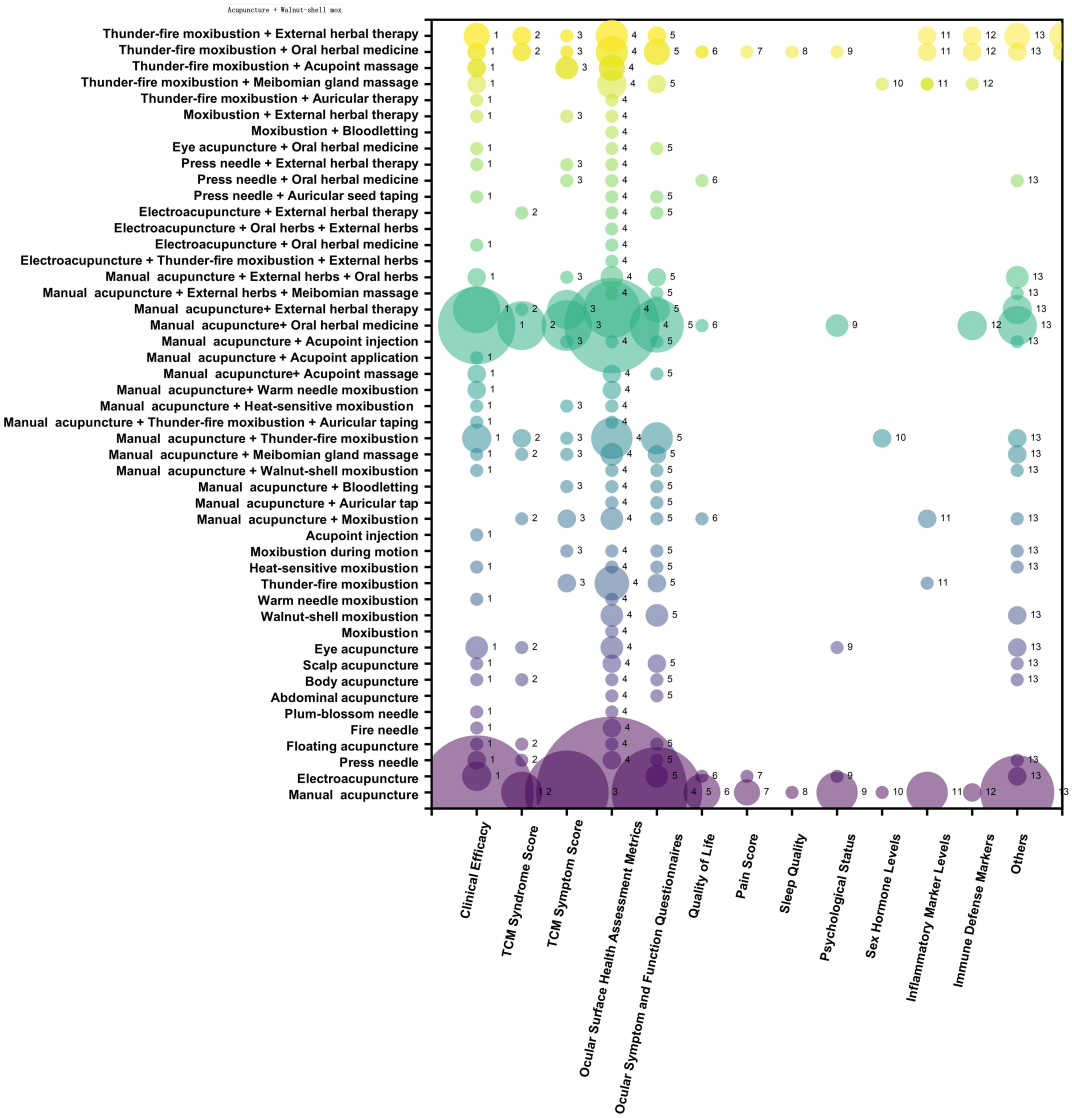
Evidence map of outcome indicators of acupuncture and moxibustion interventions for dry eye disease.

The RoB 2.0 tool recommended in the Cochrane Handbook for Systematic Reviews was used to assess the risk of bias of the 319 included RCTs on acupuncture and moxibustion for dry eye disease. The corresponding risk-of-bias plot is shown in Figure 5. The results indicated that 48 studies were rated as having a high risk of bias, mainly because random sequence generation or allocation concealment was not adequately implemented during the randomization process, and because of bias related to the selection and measurement of outcome indicators. A total of 260 studies were rated as having some concerns, most of which arose from inappropriate methods used to estimate the effect of assignment to intervention, while a smaller proportion resulted from inappropriate outcome measurement. Only 11 studies were rated as having a low risk of bias.

Specifically, for bias arising from the randomization process, 34 studies were rated as high risk, 264 as having some concerns, and 21 as low risk. For bias due to deviations from intended interventions, 3 studies were rated as high risk, 43 as having some concerns, and 273 as low risk. All studies were rated as low risk for bias due to missing outcome data. For bias in the measurement of outcomes, 12 studies were rated as high risk, 15 as having some concerns, and 292 as low risk. All studies were rated as low risk for bias in the selection of the reported result.

## 4 Discussion

### 4.1 General characteristics of the included studies

Regarding publication trends of studies on acupuncture and moxibustion interventions for dry eye disease, the number of publications showed a year-by-year increase from 2016 to 2020, suggesting that, as one of the therapeutic approaches for dry eye disease, acupuncture and moxibustion has attracted growing clinical demand and academic attention. This trend may be closely related to the rising prevalence of dry eye disease, the gradual clarification of its pathogenesis, and the development of traditional Chinese medicine. However, fluctuations in the number of publications were observed after 2020, which may be associated with a lack of innovation and increasing homogeneity in the literature over time. In terms of study scale, single-center studies with small sample sizes were found to predominate, which limits the representativeness and generalizability of the findings. Therefore, more large-scale, multicenter RCTs should be conducted in the future to improve the applicability of the evidence.

With respect to intervention duration, for reasons of convenience and cost, most studies set the treatment period at 4 weeks or less. Although such a “short-term, high-intensity” regimen may provide temporary symptomatic relief, it is difficult to produce sustained and stable effects on the underlying pathogenesis. As dry eye disease is a chronic condition, long-term and standardized follow-up should be implemented to evaluate long-term efficacy and to verify the true value of acupuncture and moxibustion in the chronic disease management of dry eye disease.

### 4.2 Intervention measures

The interventions used in studies of acupuncture and moxibustion for dry eye disease were diverse, with monotherapies mainly including acupuncture-based and moxibustion-based approaches (14). Among the acupuncture-based therapies, manual acupuncture was the most commonly used. Compared with electroacupuncture and fire acupuncture, manual acupuncture is easier to implement, less costly, and more standardized. In addition, different therapeutic effects can be achieved through flexible needling manipulations for reinforcement or reduction, such as needling Sanyinjiao (SP6) to nourish yin and reducing Taiyang (EX-HN5) to clear heat (15). In terms of efficacy, compared with artificial tears commonly used in Western medicine, manual acupuncture may offer greater advantages in controlling ocular inflammation (16). It can significantly reduce the activity of inflammatory factors such as IL-6 and TNF-*α* and provide long-lasting relief of ocular pain (17). However, due to the wide variety of acupuncture techniques and substantial differences in manipulation methods and treatment duration, there is still no widely accepted standardized intervention protocol, which limits the translation of research findings into standardized clinical pathways.

Among the moxibustion-based therapies, Leihuo moxibustion was the most frequently used. Compared with conventional moxibustion, it appears to have superior therapeutic effects. Composed of more than 20 aromatic herbs, including *Aquilaria sinensis* and Notopterygium incisum, it is considered to have stronger medicinal and thermal effects. Application around the eyes may promote meridian circulation and improve periocular blood flow, thereby effectively prolonging tear film break-up time and improving the ocular surface microenvironment (18, 19). Among the combination therapies, manual acupuncture combined with oral Chinese herbal medicine was the most commonly applied approach. This reflects the holistic view of traditional Chinese medicine through combined internal and external treatment and multi-target intervention (20). These findings suggest that the treatment of dry eye disease should be based on syndrome differentiation and pathogenesis. For example, for lung yin deficiency syndrome, modified Yangyin Qingfei Decoction may be selected to restore the dispersing function of the lung and promote the distribution of body fluids (21), while Bushen Yijing Decoction may be used for liver-kidney yang deficiency syndrome to warm yang, tonify the kidney, improve vision, and clear the liver (22). In the future, greater efforts should be made to advance individualized and dynamic TCM diagnostic and therapeutic pathways, so that the advantages of combining acupuncture with herbal medicine can be more fully realized in syndrome-based treatment.

### 4.3 Traditional Chinese medicine syndrome types involved in the included studies

The eyes depend on the nourishment of essence and qi from the Zang-fu organs. When the functions of the liver and kidney are impaired, deficiencies of qi, blood, and body fluids are likely to consume yin fluid, resulting in insufficient tear production and inadequate nourishment of the eyes (23, 24). From the perspective of syndrome type analysis, liver-kidney yin deficiency syndrome was the most frequently reported pattern. Factors such as fluctuations in sex hormones, aging, excessive use of electronic devices, chronic sleep deprivation, and increased psychological stress may all deplete liver and kidney yin (25). For this syndrome type, the fundamental therapeutic principle is to “nourish water to support wood” (26), that is, to replenish liver and kidney yin and promote the mutual generation and transformation of essence, blood, and body fluids.

In addition, in the present study, six studies involved syndrome types that were not included in the international clinical practice guidelines for traditional Chinese medicine (27), and 75.86% of the studies did not implement treatment based on TCM syndrome differentiation. This may be related to the complexity of clinical syndrome patterns and the lack of reporting standardization. Future efforts should focus on establishing more comprehensive and clinically applicable syndrome differentiation criteria for dry eye disease and on timely updating relevant guidelines, so as to improve the overall level of evidence.

### 4.4 Characteristics of outcome indicators

A total of 13 types of outcome indicators were used in the included RCTs. The findings showed that: (1) there was considerable diversity in outcome indicators, with ocular surface health assessment indicators being the most frequently used; however, the evaluation criteria involved were not standardized. (2) Subjective outcome indicators were used relatively infrequently. According to current guidelines (28), in addition to objective indicators, patient-reported subjective symptoms constitute the core manifestations and diagnostic basis of dry eye disease, and questionnaires should be further incorporated to assess specific symptoms and their impact on daily life (1). However, among the included studies, only 35.42% of the RCTs used patient-reported subjective symptoms as outcome indicators. Therefore, future studies are encouraged to strengthen the combination of subjective and objective indicators to improve the comprehensiveness of dry eye disease assessment (29). (3) At present, TCM symptom scores and TCM syndrome scores are applied relatively infrequently, largely because of the lack of scientific and standardized quantitative criteria, as well as inconsistency in specialty-specific evaluation standards (30, 31). The scoring principles and grading criteria for signs and syndrome patterns should be improved as soon as possible to facilitate the generation of high-level evidence for traditional Chinese medicine in the treatment of dry eye disease. (4) Most studies used a relatively single outcome assessment system and lacked multidimensional evaluation. Patients with dry eye disease are often accompanied by psychological problems such as anxiety and depression, which can significantly impair quality of life (6). Nevertheless, only 4.7% of the studies reported psychological status and 4.08% assessed quality of life. This suggests that when constructing multidimensional outcome sets in future studies, researchers should not only focus on ocular surface indicators but also report improvements in broader health outcomes.

### 4.5 Quality assessment of the included studies

Based on the methodological assessment conducted in this study, the following conclusions were drawn: (1) Most studies did not provide a clinical trial registration number. According to the CONSORT statement (32), RCTs should be registered on a recognized platform before study initiation in order to improve transparency and traceability. (2) Many articles failed to clearly describe the details of randomization implementation and allocation concealment strategies, thereby weakening the internal validity of the RCTs. (3) Due to the nature of the interventions, blinding of participants and practitioners was difficult to implement, and most studies also did not blind outcome assessors, which increased the risk of measurement bias. (4) The handling of loss to follow-up and missing data was often inadequate. Some studies simply excluded cases with missing data, which may have reduced the reporting quality and methodological rigor of the studies. Appropriate statistical methods for missing data should be adopted in future research to improve data reliability.

### 4.6 Limitations

This study systematically reviewed RCTs on acupuncture and moxibustion interventions for dry eye disease and integrated evidence on research trends, sample sizes, intervention measures, intervention duration, and methodological quality. Bubble plots were used to visually analyze intervention measures, TCM syndrome types, and outcome indicators, thereby providing useful evidence support for this field. Nevertheless, several limitations should be acknowledged. (1) Only RCTs were included, whereas meta-analyses, systematic reviews, and clinical guidelines were not incorporated into the analysis, which may have introduced selection bias. (2) A notable limitation of this study is that the analysis of TCM syndrome types was based on a limited and potentially selective subset of the included studies. Of the 319 included RCTs, only 77 reported TCM syndrome differentiation, and the bubble plot analysis was further limited to the 68 studies that reported a single TCM syndrome type. Consequently, this part of the analysis represents only a minority of the available evidence and may be subject to reporting bias. Furthermore, the incomplete reporting of syndrome differentiation may reflect the lack of standardized clinical application and reporting of TCM syndrome classification in dry eye disease research. Variations in syndrome differentiation criteria, diagnostic judgment, and reporting practices across studies may also have affected the consistency and comparability of the evidence. Therefore, the findings of the TCM syndrome type analysis should be interpreted cautiously, and their generalizability may be limited. (3) The tools and reporting standards for evidence mapping are still under development, which limits the comparability of the results.

## 5 Conclusion

In summary, the findings suggest a high level of clinical demand for acupuncture and moxibustion in the treatment of dry eye disease; however, the overall quality of the current evidence remains weak. Future studies should improve the evaluation framework for evidence mapping, explore the development of internationally recognized assessment scales with TCM characteristics, standardize the application of TCM syndrome types, and expand the evaluation of multidimensional health outcomes. In addition, continued efforts are needed to develop simpler, more economical, and more easily generalizable acupuncture and moxibustion protocols, so as to promote the integrative innovation of traditional acupuncture and moxibustion theories with modern technologies and fully exploit the advantages of acupuncture and moxibustion in the treatment of dry eye disease. This will provide higher-quality clinical evidence to support the use of acupuncture and moxibustion for dry eye disease.

## Data Availability

The datasets presented in this study can be found in online repositories. The names of the repository/repositories and accession number(s) can be found in the article/Supplementary material.
