# Supplementary material for: Evidence map for randomized controlled trials of treatment of acupuncture and moxibustion for dry eye disease
Source: Front Med (Lausanne). 2026 Apr 21;13:1829981. doi: 10.3389/fmed.2026.1829981 (PMC13139007; doi:10.3389/fmed.2026.1829981)
Supplement: Supplementary file 1 [file Table_1.docx]

**Supplement 2: Search strategy**

1. **PubMed database**

#1 Acupuncture [MeSH]

#2 Acupuncture OR Acupuncture therapy [Title/Abstract]

#3 #1 OR #2

#4 Acupuncture therapy [MeSH]

#5 Acupuncture therapy OR Acupuncture Treatment OR Pharmacoacupuncture Treatment OR Pharmacoacupuncture Therapy OR Acupotomy OR Acupotomies [Title/Abstract]

#6 #4 OR #5

#7 Acupuncture Points [MeSH]

#8 Acupuncture Points OR Acupoint OR Point, Acupuncture[Title/Abstract]

#9 #7 OR #8

#10Moxibustion [MeSH]

#11 Moxibustion OR Moxabustion[Title/Abstract]

#12 #10 OR #11

#13 Acupuncture, Ear[MeSH]

#14 Acupuncture, Ear OR Acupuncture, Auricular OR Auricular Acupuncture OR Ear Acupuncture[Title/Abstract]

#15 #13 OR #14

#16 #3 OR #6 OR #9 OR #12 OR #15

#17 Dry Eye Syndromes [MeSH]

#18 Dry Eye Syndromes OR Dry Eye Disease OR Dry Eye OR Evaporative Dry Eye Disease OR Evaporative Dry Eye OR Evaporative Dry Eye Syndrome[Title/Abstract]

#19 #17 OR #18

#20 #16 AND #19

**2. CNKI Chinese database**

1.针灸

2.灸法

3.取穴

4.选穴

5.穴位疗法

6.针刺

7.灸法

8.艾灸

9.雷火灸

10.悬灸

11.温灸

12.干眼

13干眼症

14.干眼病

15.眼干燥症

16.干燥性角结膜

17.角结膜干燥症

18.OR/1-11

19.OR/12-17

20.18 AND 20
